# 

**Published:** 1965-10

**Authors:** 


### Contents

A REVIEW OF LIQUOR AMNII ANALYSES DURING PREGNANCY *R. A. Branch* 61

TRANQUILLIZING AND THYMOLEPTIC DRUGS *R. E. Hemphill and J. E. Barber* 67

IS AN INTENSIVE CARE UNIT REALLY NECESSARY? *P. J. F. Baskett* 82

NEONATAL SEPTICAEMIA . . . *Clinico-Pathological Conference* 87

NOTES AND NEWS . . . . . . . . . . 95

INDEX TO VOL. 80 . . . . . . . . . . 97

